# Live‐cell imaging of the chloroplast outer envelope membrane using fluorescent dyes

**DOI:** 10.1002/pld3.462

**Published:** 2022-11-15

**Authors:** Shintaro Ichikawa, Kazuya Ishikawa, Hitoshi Miyakawa, Yutaka Kodama

**Affiliations:** ^1^ Center for Bioscience Research and Education Utsunomiya University Tochigi Japan; ^2^ Graduate School of Regional Development and Creativity Utsunomiya University Tochigi Japan

**Keywords:** chloroplast, chloroplast outer envelope membrane, confocal laser scanning microscopy, fluorescent dye, live‐cell imaging, Nile red, rhodamine B, staining, sub‐organellar compartment, various plant species

## Abstract

Chloroplasts are organelles composed of sub‐organellar compartments—stroma, thylakoids, and starch granules—and are surrounded by outer and inner envelope membranes (OEM and IEM, respectively). The chloroplast OEM and IEM play key roles not only as a barrier separating the chloroplast components from the cytosol but also in the interchange of numerous metabolites and proteins between the chloroplast interior and the cytosol. Fluorescent protein markers for the chloroplast OEM have been widely used to visualize the outermost border of chloroplasts. However, the use of marker proteins requires an established cellular genetic transformation method, which limits the plant species in which marker proteins can be used. Moreover, the high accumulation of OEM marker proteins often elicits abnormal morphological phenotypes of the OEM. Because the OEM can currently only be visualized using exogenous marker proteins, the behaviors of the chloroplast and/or its OEM remain unknown in wild‐type cells of various plant species. Here, we visualized the OEM using live‐cell staining with the fluorescent dyes rhodamine B and Nile red in several plant species, including crops. We propose rhodamine B and Nile red as new tools for visualizing the chloroplast OEM in living plant cells that do not require genetic transformation.

**Significance Statement:**

We established a live‐cell imaging method to visualize the chloroplast outer envelope membrane by staining living cells with fluorescent dyes. This method does not require genetic transformation and allows the observation of the chloroplast outer envelope membrane in various plant species.

## 1 INTRODUCTION

Chloroplasts are unique organelles present in plants and green algae that are derived from endosymbiotic cyanobacteria engulfed by a non‐photosynthetic eukaryote host (Howe et al., 2008; Larkum et al., 2007). In chloroplasts, sub‐organellar compartments including thylakoids, starch granules, and the stroma matrix functionally interact with each other and contribute to various metabolic pathways such as photosynthesis (Pfannschmidt, 2003; Poolman et al., 2004; Taniguchi & Miyake, 2012). These sub‐organellar compartments and matrix are encapsulated by the double‐layered chloroplast envelope membrane: the outer and inner envelope membranes (OEM and IEM, respectively) (Staehelin, 2003). The OEM and IEM act as an organellar barrier separating the inside of the chloroplast from the cytosol, preventing ions, metabolites, and proteins from being transported passively across the chloroplast envelope. During multiple metabolic processes in chloroplasts, a number of molecules are exchanged between the chloroplast and cytosol via transporters that localize at the OEM and IEM (Inoue, 2007; Rolland et al., 2012; Smith & Zeeman, 2020). In addition, a large proportion of the proteins localizing in the chloroplast are encoded by the nuclear genome. Therefore, almost all chloroplast proteins are imported from the cytosol into the chloroplast via translocons at the OEM and IEM (TOC and TIC, respectively) (Jarvis, 2008; Kessler & Schnell, 2006). The OEM and IEM perform essential functions during molecular transport in plant cells.

Because the OEM is the outermost layer of the chloroplast, fluorescent protein (FP) markers associated with the chloroplast OEM have been used to examine chloroplast morphology (Duy et al., 2007; Li et al., 2020; Oikawa et al., 2008; Shang et al., 2010; Spitzer et al., 2015; Tanaka et al., 2017; Yoshizumi et al., 2018). For example, FPs fused to OUTER ENVELOPE PROTEIN7 (OEP7), CHLOROPLAST UNUSUAL POSITIONING1 (CHUP1), and TOC64 are used as OEM marker proteins (Breuers et al., 2012; Lee et al., 2001; Oikawa et al., 2008). However, such OEM marker proteins can only be deployed in the subset of plant species for which transformation methods have been developed. Moreover, overexpression of genes encoding OEM marker proteins often induces abnormal phenotypes, such as membrane protrusion and chloroplast aggregation (Breuers et al., 2012; Oikawa et al., 2008). Owing to the technical limitations associated with OEM marker proteins, the behaviors of chloroplasts and/or their OEM remain to be determined in wild‐type cells of various plant species.

In this study, we established that the chloroplast OEM can be visualized in living cells of multiple plant species by simple staining with the fluorescent dyes rhodamine B (RhB) and Nile red (NR). Transformation techniques are not required for our dye‐staining method, and the chloroplast OEM can be visualized via fluorescence microscopy within 10 min of staining.

## 2 RESULTS

### 2.1 RhB and NR stain the chloroplast envelope

**FIGURE 1:**
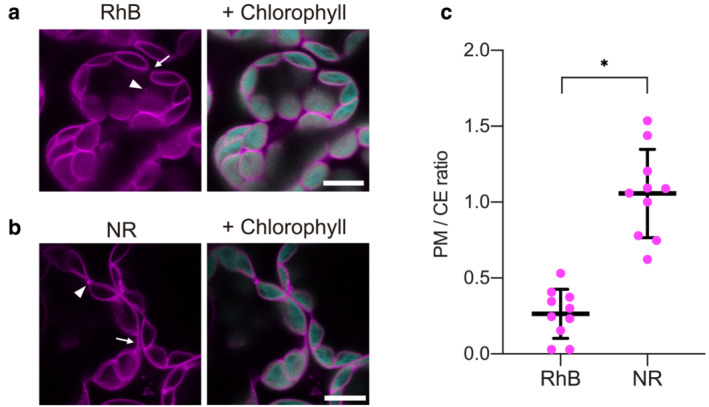
Visualization of the chloroplast envelope using rhodamine B and Nile red staining. (a,b) Leaf cells of *Nicotiana benthamiana* stained with (a) rhodamine B (RhB) or (b) Nile red (NR). Arrows indicate the positions of the plasma membrane, and arrowheads indicate non‐specific signal. Scale bars, 10 μm. (c) Fluorescence intensity ratio between the plasma membrane and the chloroplast envelope (PM/CE ratio). RhB and NR fluorescence intensities were determined at the plasma membrane (PM) and the chloroplast envelope (CE), and the mean and standard deviation of the PM/CE ratio were calculated (*n* = 10). An asterisk indicates a significant difference (Student's *t*‐test, *p* < .05).

In a previous study using isolated chloroplasts, RhB and NR staining were used to distinguish between intact and disrupted chloroplasts; only intact chloroplasts were visualized as spherical shapes using the two fluorescent dyes, indicating that RhB and NR possibly stain the OEM and/or IEM of isolated chloroplasts (An et al., 2021). RhB and NR have also been reported to be membrane‐permeable dyes (Greenspan et al., 1985; Grolig & Wagner, 1987; Halim & Webley, 2015; Mottram et al., 2012). In agreement with these observations, we confirmed the uptake of these fluorescent dyes across the plasma membrane of *Drosophila melanogaster* Schneider 2 (S2) cells (Figure S1). Given the characteristics of RhB and NR with respect to the chloroplast envelope and plasma membrane, we suspected that RhB and NR might be applicable to visualizing the chloroplast OEM and/or IEM in living plant cells. To test our hypothesis, we treated *Nicotiana benthamiana* leaf cells with 1 μM RhB or NR for 10 min. We then observed the stained cells using confocal laser scanning microscopy (CLSM), which revealed that the chloroplast periphery is stained by both RhB and NR in these living cells (Figure 1a,b). Although we observed the RhB signal at the chloroplast envelope (Figure 1a), we detected the NR signal at the chloroplast envelope and at the plasma membrane (Figure 1b). We also noticed additional cytoplasmic punctate signals upon individual staining with both RhB and NR, but these signals were negligible because of their very low signal intensity (Figure 1a,b). To investigate the specificity of chloroplast envelope staining with RhB and NR, we quantified the ratio of fluorescence intensity at the plasma membrane to that at the chloroplast envelope (PM/CE ratio) in *N. benthamiana* cells treated with RhB or NR (Figure 1c). The mean PM/CE ratio of NR was approximately 1.06 (Figure 1c), suggesting that the fluorescence intensity at the chloroplast envelope was comparable with that at the plasma membrane. By contrast, the mean PM/CE ratio of RhB was about 0.27 (Figure 1C), indicating that RhB stains the chloroplast envelope rather than the plasma membrane. RhB therefore seems to be more suitable for visualizing the chloroplast envelope in living plant cells.

### 2.2 RhB and NR can be used to visualize the chloroplast envelope in a variety of plant species

**FIGURE 2:**
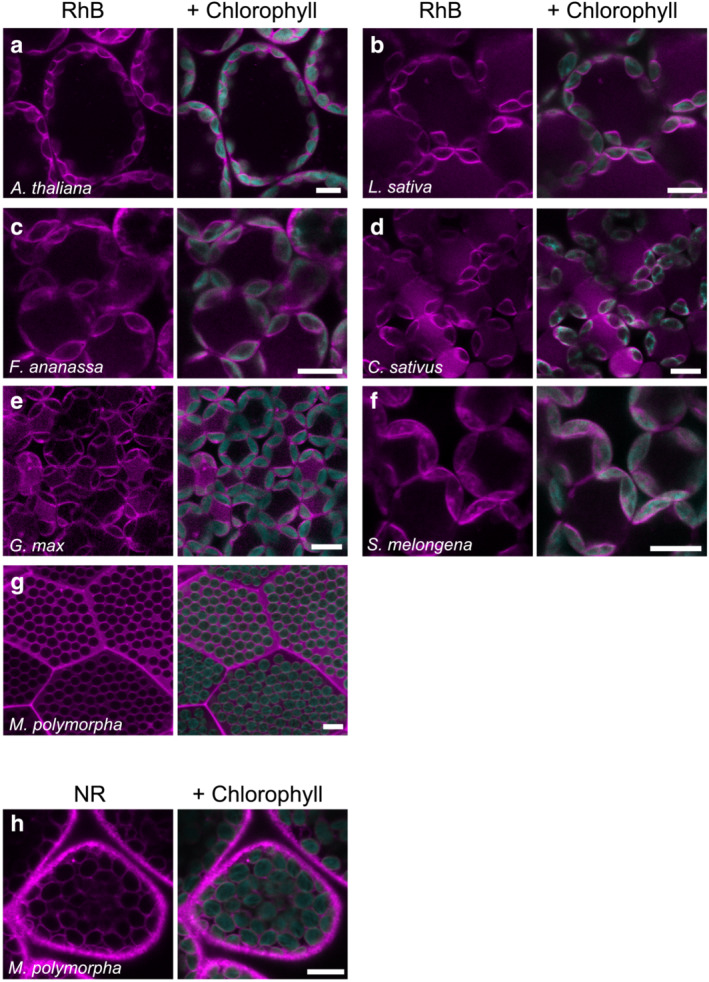
RhB staining of the chloroplast envelope in several plant species. (a) thale cress ( *Arabidopsis thaliana* ), (b) lettuce ( *Lactuca sativa* ), (c) strawberry (*Fragaria × ananassa*), (d) cucumber ( *Cucumis sativus* ), (e) soybean ( *Glycine max* ), (f) eggplant ( *Solanum melongena* ), and (g) liverwort ( *Marchantia polymorpha* ) cells stained with 1 μM RhB. (h) *M. polymorpha* cells stained with 1 μM NR. Scale bars, 10 μm.

We next examined whether RhB and NR can stain the chloroplast envelope in various plant species: *Arabidopsis thaliana* (thale cress), *Lactuca sativa* (lettuce), *Fragaria × ananassa* (strawberry), *Cucumis sativus* (cucumber), *Solanum melongena* (eggplant), *Glycine max* (soybean), and *Marchantia polymorpha* (liverwort). When cells were treated with 1 μM RhB, we determined that the chloroplast envelope is stained specifically in all species except *M. polymorpha* (Figure 2a–f). The observed staining pattern was similar to that of *N. benthamiana* leaf cells (Figure 1a). By contrast, we detected the RhB signal in the cytosol in *M. polymorpha*, but not at the chloroplast envelope (Figure 2g, and Figure S2a,b). Importantly, we successfully visualized the chloroplast envelope and the plasma membrane when we stained *M. polymorpha* with 1 μM NR (Figure 2h), yielding a pattern similar to that in *N. benthamiana* (Figure 1b). We also observed a similar staining pattern by NR in thale cress, lettuce, strawberry, and cucumber (Figure S2). NR is likely to be applicable to a wide range of plant species but has lower specificity for the chloroplast envelope than RhB (Figures 2h and S2). Although there were exceptions (e.g., *M. polymorpha*), RhB appears to preferentially stain the chloroplast envelope in various living plant cells.

We also tested whether RhB and NR can stain the envelope of non‐photosynthetic plastids. To this end, we incubated *A. thaliana* root cells with 1 μM RhB or NR and observed that RhB slightly stains the plastid envelope, whereas NR showed no staining of the plastid envelope (Figure S3). We further investigated whether RhB and NR might be applicable for the visualization of the chloroplast envelope in green algae. When *Chlamydomonas reinhardtii* and *Euglena gracilis* cells were incubated with RhB or NR, we failed to visualize either fluorescent dye at the chloroplast envelope in either alga (Figure S4).

### 2.3 RhB stains the chloroplast outer membrane

**FIGURE 3:**
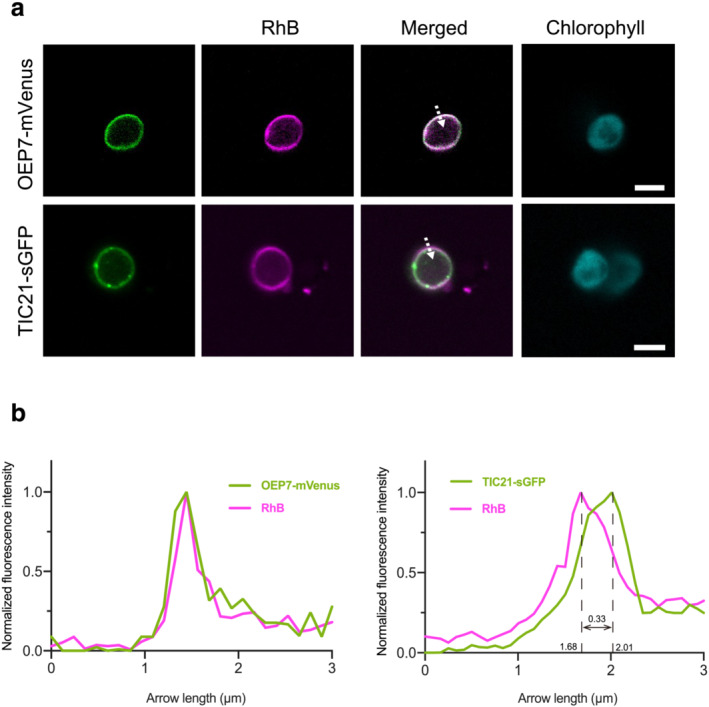
Analysis of RhB staining in the chloroplast envelope using isolated chloroplasts. (a) Isolated chloroplasts from *Nicotiana benthamiana* leaves expressing *OEP7‐mVenus* or *TIC21‐sGFP*, stained with 1 μM RhB. Scale bars, 5 μm. Dashed arrows indicate the region (3 μm in length) used to measure the fluorescence intensity profile in (b). (b) Profile analysis of RhB with OEP7‐mVenus (left graph) and TIC21‐sGFP (right graph) fluorescence intensities. Fluorescence intensity was measured on a line [dashed arrows in (a)] passing through the outside and inside of a chloroplast and normalized to the highest fluorescence intensity value along the line.

To determine whether RhB stains the chloroplast OEM or IEM, we isolated chloroplasts from *N. benthamiana* leaves expressing the OEM or IEM FP marker *OEP7‐mVenus* or *TIC21‐sGFP*, respectively (Lee et al., 2001; Shang et al., 2010; Teng et al., 2006), and stained the isolated chloroplasts with RhB. When we observed the stained chloroplasts using CLSM, the RhB signal completely coincided with that of OEP7‐mVenus, whereas the RhB signal only partially overlapped with that of TIC21‐sGFP (Figure 3a). A quantification of fluorescence intensities of the collected images (Figure 3a) revealed that the fluorescence peak of RhB matches that of OEP7‐mVenus, but not TIC21‐sGFP (Figure 3b). We estimated the distance between the fluorescence peaks of RhB and TIC21‐sGFP to be 0.33 μm (Figure 3b), consistent with the fluorescence images (Figure 3a), indicating that RhB stains the OEM of isolated chloroplasts.

**FIGURE 4:**
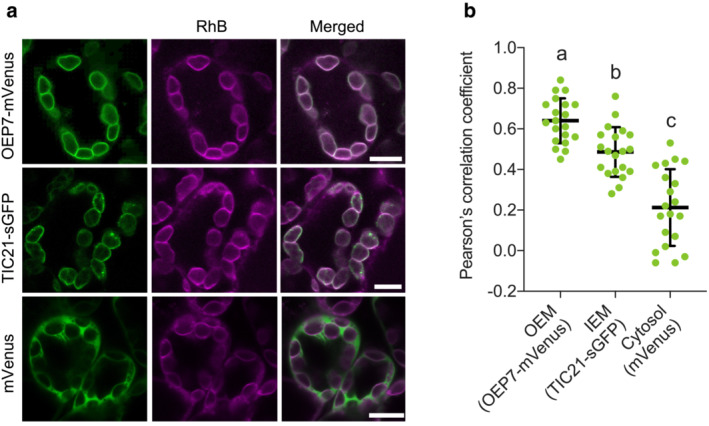
Colocalization analysis of RhB with the OEM and IEM visualized using fluorescent proteins. (a) *Nicotiana benthamiana* leaf cells transiently expressing *OEP7‐mVenus*, *TIC21‐sGFP*, or *mVenus*, stained with 1 μM RhB. Scale bars, 5 μm. (b) Colocalization analysis of RhB signal with OEP7‐mVenus (OEM), TIC21‐sGFP (IEM), and mVenus (cytosol). The mean values of Pearson's correlation coefficient for the OEM, IEM, and cytosol were 0.64, 0.49, and 0.21, respectively. For colocalization analysis, the mean and standard deviation of the Pearson's correlation coefficient were determined and compared (*n* = 20). Different lowercase letters indicate significant differences (Tukey's multiple comparison test, *p* < .05).

To confirm that RhB stains the OEM in living cells, we carried out a colocalization analysis with OEP7‐mVenus or TIC21‐sGFP. As a control, we used mVenus alone as a cytosolic FP marker. Using *Agrobacterium*‐mediated infiltration, we transiently expressed *mVenus* alone, *OEP7‐mVenus*, or *TIC21‐sGFP* in *N. benthamiana* cells, followed by staining with RhB and image acquisition by CLSM. We observed that the RhB signal visibly colocalizes with that of both OEP7‐mVenus and TIC21‐sGFP, but not with that of cytosolic mVenus (Figure 4a). To assess whether RhB stains the chloroplast OEM or IEM, we performed a correlation analysis via Pearson's correlation coefficient, which determines the degree of colocalization by calculating the fluorescence intensity ratio using two‐channel merged images (Dunn et al., 2011) (Figure 4b). The mean of Pearson's correlation coefficient between RhB staining and OEP7‐mVenus at the OEM was significantly higher than that with TIC21‐sGFP at the IEM or that of mVenus alone in the cytosol. Together with the results using isolated chloroplasts (Figure 3a,b), we concluded that RhB fluorescently labels the chloroplast OEM in living plant cells.

## 3 DISCUSSION

In the present study, we demonstrated that the OEM could be visualized in cells from various plant species by staining live samples with the dye RhB for 10 min, thus bypassing the need for genetic transformation. Our RhB‐based live‐cell imaging of the OEM shed light on the innate characteristics of chloroplasts in multiple plant species.

RhB and NR are known to bind to lipids (Gao et al., 2006; Krishnamoorthy, 1998; Mukherjee et al., 2007; Sackett et al., 1990; Verdin et al., 2005). Considering these previous studies and our results, we hypothesize that RhB and NR might bind to lipids of the chloroplast envelope. Our CLSM experiments revealed that the binding specificity for the chloroplast envelope was higher for RhB than for NR; RhB specifically stained the chloroplast envelope, whereas NR stained not only the chloroplast envelope but also the plasma membrane. Because the lipid composition of the plasma membrane is different from that of the chloroplast OEM (Jouhet et al., 2007), RhB might bind to specific lipids that comprise or are abundant in the chloroplast OEM. Remarkably, the lipid composition of the chloroplast envelope varies among plant species and algae (Poincelot, 1976; Schwertner & Biale, 1973; Thompson, 1996). Because RhB did not stain the OEM in *M. polymorpha* (Figure 2h), the amount of the specific lipids that are bound by RhB may be lower in *M. polymorpha* than in the other plant species tested. In the case of NR, the plastid envelope of *A. thaliana* root cells was not labeled by the dye (Figure S4). The amount of the specific lipids bound by NR may thus be lower in the root plastid than in leaf chloroplasts. Given that neither RhB nor NR stained the chloroplast envelope in algae, we speculate that the chloroplast envelope in algae does not contain the specific lipids bound by RhB and NR. If these specific lipids are identified in the future, it may be possible to predict the plant species and plastid types whose OEM can be stained by RhB and/or NR.

When we observed the OEM using RhB and NR, we also observed cytoplasmic punctate signals (Figure 1a,b). Previous studies reported that RhB can stain mitochondria in the nematode *Caenorhabditis elegans* (Mottram et al., 2012), and NR was used to stain lipid droplets (Greenspan et al., 1985). If RhB and NR visualize the OEM by binding to specific lipids, other subcellular membranes containing the same lipids might be stained. However, based on our imaging results, the amount of the specific lipids bound by RhB and NR appears to be lower in other subcellular membranes than in the chloroplast OEM.

Previous studies have illustrated how the OEM and IEM are separated by 10–20 nm in *C. reinhardtii* and 5–10 nm in *A. thaliana* using cryo‐electron tomography and transmission electron microscopy, respectively (Engel et al., 2015; Inoue, 2007; Keegstra et al., 1984). Based on this information, the OEM and IEM should not be distinguishable using CLSM. Indeed, the CLSM crossing resolution is determined by the full width at half maximum (FWHM) parameter. If the specimen is uniformly irradiated by the laser, the FWHM is calculated using the following formula (Wang et al., 2003): FWHM = 0.37λ/NA (λ being the wavelength and NA the numerical aperture). Under our observation conditions, the NA value of the ×63 magnification water lens in the SP8X confocal microscope system was 1.2 (Leica, HC PL APO 63×/1.20 W CORR CS2), and the peaks of the emission wavelengths for sGFP, mVenus, and RhB were 510, 540, and 600 nm, respectively. Based on this information, the corresponding FWHMs are 157, 167, and 185 nm, respectively. However, in this study, we successfully detected distinct signals emanating from the OEM and IEM in the same living plant cell using CLSM and Pearson's correlation coefficient (Figure 4). There are two possibilities that may explain the detection of separate OEM‐ and IEM‐derived fluorescence signals with the constraints of CLSM resolution. First, the distance between the two membranes in living plant cells might be partly larger than the CLSM resolution. Importantly, in the case of isolated chloroplasts (Figure 3), the distance between the OEM and IEM increased (approximately 330 nm) (Figure 3b). Partial separation of the two envelope membranes might therefore be easily observed in isolated chloroplasts. Second, the topological difference between RhB and TIC21‐sGFP should be considered; RhB localizes to the cytosolic face of the OEM, whereas sGFP fused to TIC21 resides on the stromal face of the IEM. Such a topology may increase the distance between these two fluorescent signals, even if their molecular size is in nanometers.

Live‐cell imaging analysis is a powerful tool for understanding intracellular phenomena in plants. Although FPs are widely used for visualizing organelles and sub‐organellar compartments in living cells, genetic transformation is a prerequisite for the expression of transgenes encoding these FPs in plant cells. Transformation methods have yet to be developed for many plant species, including crops. By contrast, an imaging method based on an incubation with fluorescent dyes does not require genetic transformation, making cell staining with a fluorescent dye applicable to a variety of plant species. Fluorescent dyes are currently available that stain organelles/sub‐organelles such as the nucleus, mitochondria, and the endoplasmic reticulum (Lee et al., 2013; Maxwell et al., 1999; Meadows & Potrykus, 1981). In the present study, we established that RhB and NR can be used to visualize the chloroplast OEM in various living cells, making them part of the collection of fluorescent dyes available for visualizing organelles/sub‐organelles in plant cells. However, fluorescent dyes have not yet been identified to visualize other chloroplast sub‐compartments such as the IEM, the stroma, or starch granules. Expanding the catalog of fluorescent dyes for live‐cell imaging of organelles/sub‐organelles is our next challenge.

## 4 MATERIALS AND METHODS

### 4.1 Plant materials and growth and cell culture conditions

*Nicotiana* *benthamiana* plants were grown on half‐strength Murashige and Skoog (MS) medium for 12 days under a 16‐h‐light (approximately 50 μmol photons m^−2^ s^−1^)/8‐h‐dark photoperiod at 25°C before being transplanted to soil. For experiments, 30‐ to 40‐day‐old *N*. *benthamiana* plants were used. *Arabidopsis* *thaliana* (Columbia‐0 accession) was grown on half‐strength MS medium for 12 days under continuous white light (approximately 50 μmol photons m^−2^ s^−1^) at 22°C. After transfer to soil, 25‐day‐old plants were used for leaf cell observations. When plastids in *A*. *thaliana* root cells were observed, *A*. *thaliana* roots from plants grown on half‐strength MS medium for 2–3 weeks under continuous white light at 22°C were used. The liverwort *M*. *polymorpha* (Takaragaike‐1 accession) was grown on half‐strength Gamborg's B5 medium under continuous white light at 22°C, and 1‐day‐old gemmalings were used. Lettuce (*L*. *sativa*) and cucumber (*C*. *sativus*) were grown on soil at 22 or 25°C, respectively, for 30 days. *Chlamydomonas* *reinhardtii* was grown in Tris‐acetate phosphate medium under a 16‐h‐light/8‐h‐dark photoperiod at 25°C and used for staining. *Euglena* *gracilis* (strain Z) was grown in Cramer–Myers medium (Cramer & Myers, 1952) containing 0.1% (v/v) ethanol under a 16‐h‐light/8‐h‐dark photoperiod at 25°C and used for staining. Strawberry (*Fragaria* × *ananassa*), soybean (*G*. *max*), and eggplant (*S*. *melongena*) were harvested in Sakura City, Tochigi, Japan. *Drosophila* *melanogaster* S2 cells were cultured in Schneider's *D*. *melanogaster* medium containing 10% (v/v) fetal bovine serum and grown at 28°C (Miyakawa et al., 2018).

### 4.2 Plasmid cloning and construction

All primers used for plasmid construction are listed in Table S1. The DNA fragment encoding TIC21 (At2g15290) was PCR‐amplified from *A. thaliana* genomic DNA using the primers TIC21‐SalI‐F and TIC21‐EcoRV‐R (Table S1). The DNA fragment for *mVenus* was PCR‐amplified from a synthetic double‐stranded DNA (Integrated DNA Technologies) encoding mVenus (Venus‐A206K) using the primers mVenus‐SalI‐F and mVenus‐EcoRV‐R (Table S1). To create a fusion gene encoding the N‐terminal 50 amino acids of OEP7 (At3g52420) with mVenus (*OEP7‐mVenus*), the DNA fragment for *OEP7* was PCR‐amplified from pDONR207‐OEP7 (Tanaka et al., 2017) using the primers OEP7‐SalI‐F and OEP7‐linker‐R (Table S1), and the DNA fragment for *mVenus* was PCR‐amplified using the primers Linker‐mVenus‐F and mVenus‐EcoRV‐R (Table S1). The resulting two fragments were joined by recombinant PCR, generating the *OEP7‐mVenus* fragment. *TIC21*, *mVenus*, and *OEP7‐mVenus* fragments were cloned into the pENTR1A vector (Invitrogen) at the *Sal*I and *Eco*RV sites using an In‐Fusion HD Cloning Kit (Clontech). The *TIC21* fragment cloned in the pENTR1A vector was recombined into the pGWB405 binary vector, which is engineered to express cloned genes under the control of the cauliflower mosaic virus (CaMV) 35S promoter as a C‐terminal sGFP (GFP‐S65T) fusion protein (Nakagawa et al., 2007). The *mVenus* and *OEP7‐mVenus* fragments cloned in the pENTR1A vector were recombined into the pGWB602 binary vector, which is engineered to express cloned genes under the control of the CaMV 35S promoter (Nakagawa et al., 2007). Recombination into the binary vectors was performed using the Gateway LR reaction system (Invitrogen). The resulting pGWB405‐TIC21, pGWB602‐mVenus, and pGWB602‐OEP7‐mVenus plasmids were transformed into *Agrobacterium tumefaciens* strain GV2260. Positive *A. tumefaciens* transformants were used for transient infiltration experiments.

### 4.3 *Agrobacterium*‐mediated infiltration of *N. benthamiana* leaves

*Agrobacterium*‐mediated infiltration was performed according to a previous study (Kapila et al., 1997). Positive *A*. *tumefaciens* transformants carrying the plasmids of interest were cultured in 2 ml LB medium at 28°C for 24 h in a shaking incubator. After culture, 1 ml of the culture was transferred to a tube and centrifuged at 4000 *g* for 3 min at 20°C. After removing the supernatant, the *A. tumefaciens* pellet was resuspended in infiltration buffer (10 mM MgCl_2_ and 10 mM MES‐KOH pH 5.7). The cell suspension was adjusted to OD_600_ = 2.0 and used for infiltration.

### 4.4 Chloroplast isolation

Chloroplast isolation was performed according to previous studies (Cline et al., 1985; Fan et al., 1999). *Nicotiana benthamiana* leaves (0.4 g) in 5 ml of chloroplast preparation (CP) buffer (30 mM HEPES‐KOH pH 7.5, 0.33 M sorbitol, 2 mM EDTA pH 8.0, and 0.1% [w/v] BSA) were finely chopped using a cutter on ice, and the chopped sample was filtered through two layers of Miracloth (Merck). The filtrate containing chloroplasts was centrifuged in a swing rotor at 1000 *g* for 10 min at 4°C. After removing the supernatant, CP buffer was added to the chloroplast pellet, and the pellet was resuspended gently in CP buffer. Percoll buffer (80% [w/v] Percoll in CP buffer) was added to the chloroplast suspension to adjust the Percoll density to 20% (w/v). The adjusted suspension was gently added to the top of a Percoll gradient solution consisting of 40% (w/v) and 80% (w/v) Percoll buffer. The resulting Percoll solution was centrifuged at 800 *g* for 20 min at 4°C to separate intact and broken chloroplasts. The lower layer representing intact chloroplasts was collected. To change the solvent, 1 ml CP buffer was added to the intact chloroplasts and centrifuged at 800 *g* and 4°C for 10 min. After removing the supernatant, CP buffer was added to the chloroplasts.

### 4.5 Observation conditions and confocal microscopy

Leaf discs (2.0 mm in diameter) were excised from plants of all species using a hole puncher (Natsume Seisakusho, KN‐291‐2). To stain leaf cells with RhB or NR, leaf discs were incubated in 1 μM RhB or NR dissolved in water for 10 min. To stain *A. thaliana* root cells, detached roots were incubated in 1 μM RhB or NR dissolved in water for 10 min. *Chlamydomonas* *reinhardtii* and *E. gracilis* were stained with 1 μM RhB or NR dissolved in 0.1% (w/v) agarose and directly mounted onto glass slides for observation. To stain isolated chloroplasts with RhB, isolated chloroplasts were mounted onto glass slides in 1 μM RhB dissolved in water. Schneider's *D. melanogaster* medium was removed from S2 cells cultured in a glass‐bottom plate (Matsunami, D11140H); 1 μM RhB or NR in phosphate‐buffered saline (PBS) was then added to the S2 cells and incubated for 10 min. After removing RhB or NR, the stained S2 cells were washed twice with PBS and observed by CLSM. The fluorescence of sGFP, mVenus, RhB, NR, and chlorophyll was observed using an SP8X confocal microscope system (Leica Microsystems) with an objective lens (Leica, HC PL APO 63×/1.20 W CORR CS2). The pinhole size was set to 98 μm. sGFP was excited at 488 nm from a white light laser (WLL) and detected at 500–550 nm. mVenus was excited at 514 nm from a WLL and detected at 525–560 nm. RhB and NR were excited at 570 nm from a WLL and detected at 590–640 nm. Chlorophyll autofluorescence was excited at 570 nm and detected at 680–720 nm. To minimize bleed‐through by chlorophyll fluorescence in the sGFP, mVenus, RhB, and NR images, the time‐gated method was used with a detection time of 0.5–1.2 ns (Kodama, 2016).

### 4.6 Analysis using the PM/CE ratio

To determine the PM/CE ratio, RhB and NR fluorescence intensities at the plasma membrane (PM) and the chloroplast envelope (CE) were quantified. Fluorescence intensities were randomly measured at 10 positions of the CE and the PM each (10 pixels total each) located near the chloroplast being measured in a cell. Mean values of the fluorescence intensities at the CE and PM were calculated, and the PM/CE ratio was determined. This measurement was repeated to obtain 10 PM/CE ratios from 10 different cells, and the mean and standard deviation were calculated for statistical analysis using Student's *t*‐test.

### 4.7 Colocalization analysis using Pearson's correlation coefficient

For this analysis, original images were captured with a resolution of 1024 × 1024 pixels, and the pinhole size was set to 60 μm. The original images were cropped to 200 × 200‐pixel images showing fluorescence of FP (OEP7‐mVenus or TIC21‐sGFP) and RhB. Colocalization analysis was performed using the Coloc 2 plug‐in installed in ImageJ Fiji (https://imagej.net/software/fiji/). Colocalization analysis was carried out using cropped images according to a previous study (Costes et al., 2004), and Pearson's correlation coefficient were calculated as the ratio of each channel fluorescence intensity per pixel (total 200 × 200 pixels). Following the threshold determined by Costes, the above‐threshold Pearson's correlation coefficient was adopted (Barlow et al., 2010).

## CONFLICT OF INTEREST

The authors declare that they have no competing interests.

## AUTHOR CONTRIBUTIONS

S.I. and Y.K. planned the experiments. S.I. performed most of the experiments. K.I. constructed the plasmids. H.M. prepared the *D. melanogaster* cells. S.I. and Y.K. performed the data analysis. S.I. and Y.K. wrote the manuscript. S.I. and Y.K. prepared the figures and tables. All authors discussed the results and revised the manuscript.

## Supporting information


**Figure S1.** **Membrane permeability of RhB and NR.**
*Drosophila* *melanogaster* S2 cells treated with RhB (A) or NR (B) to confirm membrane permeability. Scale bars, 10 μm.
Figure S2. NR staining of the chloroplast envelope in several plant species.
Leaf cells of (A) thale cress ( *A. thaliana* ), (B) lettuce ( *L. sativa* ), (C) strawberry (*F. × ananassa*), and (D) cucumber ( *C. sativus* ) stained with 1 μM NR. Scale bars, 10 μm.
Figure S3. RhB and NR staining in *Arabidopsis* *thaliana* root.
*Arabidopsis* *thaliana* roots were treated with 1 μM RhB (A) or 1 μM NR (B). Scale bars, 20 μm.
Figure S4. RhB and NR staining in algae
*Chlamydomonas reinhardtii* (A and B) and *Euglena gracilis* (C and D) were treated with 1 μM RhB or NR. Scale bars, 10 μm (A and B) or 20 μm (C and D).
Table S1. Primers used in this study.

## Data Availability

All relevant data can be found within the manuscript and its supporting materials.
