# Supplementary material for: Live‐cell imaging of the chloroplast outer envelope membrane using fluorescent dyes
Source: Plant Direct. 2022 Nov 15;6(11):e462. doi: 10.1002/pld3.462 (PMC9666008; doi:10.1002/pld3.462)
Supplement: Supplementary file 1 — Figure S1. Membrane permeability of RhB and NR. Drosophila melanogaster S2 cells treated with RhB (A) or NR (B) to confirm membrane permeability. Scale bars, 10 μm. Figure S2. NR staining of the chloroplast envelope in several plant species. Leaf cells of (A) thale cress ( A. thaliana ), (B) lettuce ( L. sativa ), (C) strawberry (F. × ananassa), and (D) cucumber ( C. sativus ) stained with 1 μM NR. Scale bars, 10 μm. Figure S3. RhB and NR staining in Arabidopsis thaliana root. Arabidopsis thaliana roots were treated with 1 μM RhB (A) or 1 μM NR (B). Scale bars, 20 μm. Figure S4. RhB and NR staining in algae Chlamydomonas reinhardtii (A and B) and Euglena gracilis (C and D) were treated with 1 μM RhB or NR. Scale bars, 10 μm (A and B) or 20 μm (C and D). Table S1. Primers used in this study. [file PLD3-6-e462-s001.pdf]

Supporting Information

Live-cell imaging of the chloroplast outer envelope membrane using fluorescent dyes

Shintaro Ichikawa[1,2], Kazuya Ishikawa[1], Hitoshi Miyakawa[1,2], Yutaka Kodama[1,2*]

[1]Center for Bioscience Research and Education, Utsunomiya University, Tochigi 321-8505, Japan
[2]Graduate School of Regional Development and Creativity, Utsunomiya University, Tochigi 321-8505, Japan

[*]e-mail: kodama@cc.utsunomiya-u.ac.jp

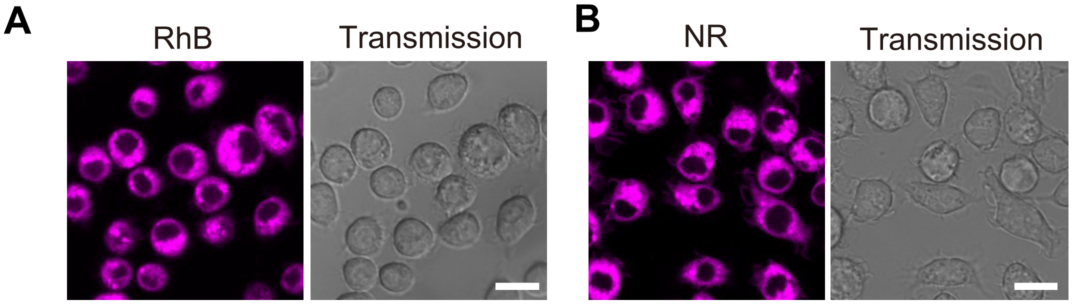

Figure S1. Membrane permeability of RhB and NR.
Drosophila melanogaster S2 cells treated with RhB (A) or NR (B) to confirm membrane permeability. Scale bars, 10 μm.

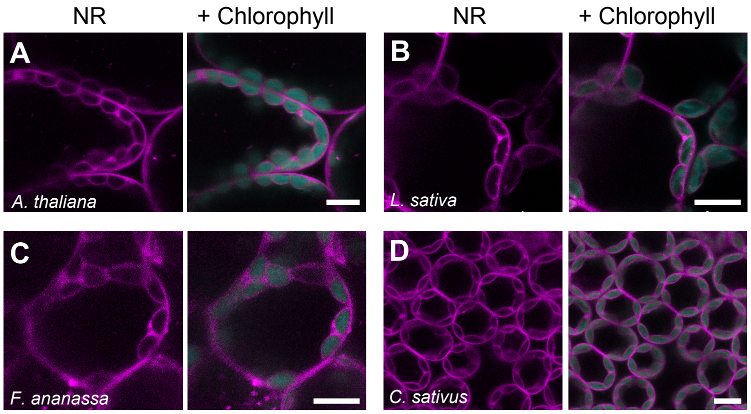

Figure S2. NR staining of the chloroplast envelope in several plant species.
Leaf cells of (A) thale cress (*A. thaliana*), (B) lettuce (*L. sativa*), (C) strawberry (*F.* × *ananassa*), and (D) cucumber (*C. sativus*) stained with 1 µM NR. Scale bars, 10 µm.

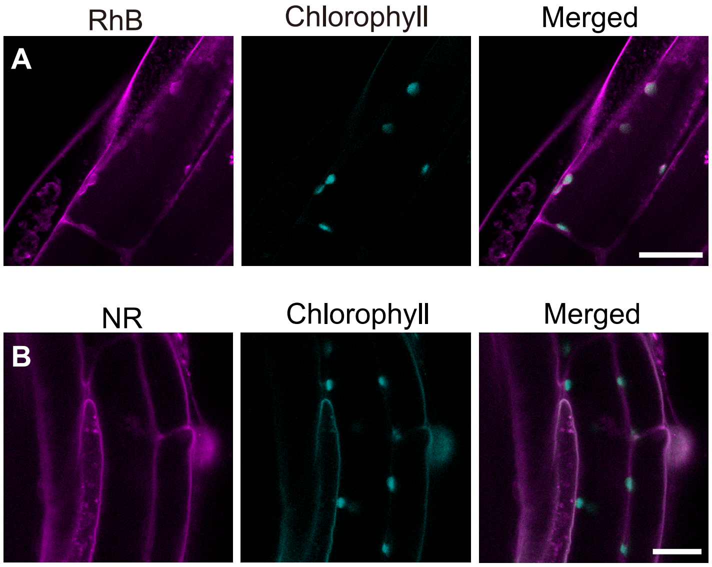

Figure S3. RhB and NR staining in *Arabidopsis thaliana* root.
Arabidopsis thaliana roots were treated with 1 µM RhB (A) or 1 µM NR (B). Scale bars, 20 µm.

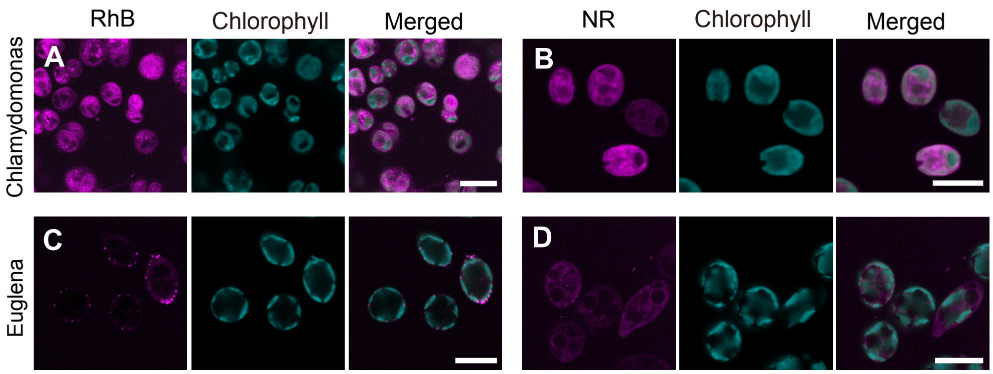

Figure S4. RhB and NR staining in algae
Chlamydomonas reinhardtii (A and B) and *Euglena gracilis* (C and D) were treated with 1 µM RhB or NR. Scale bars, 10 µm (A and B) or 20 µm (C and D).

Table S1. Primers used in this study.

Primer name	Primer sequence (5′ to 3′)
TIC21-SalI-F	AACCAATTCAGTCGACATGCAATCACTACTCTTG
TIC21-EcoRV-R	AAGCTGGGTCTAGATATCCAGCAACCTTAGGAACTAC
mVenus-SalI-F	AACCAATTCAGTCGACATGGTGAGCAAGGGCGAG
mVenus-EcoRV-R	AAGCTGGGTCTAGATATCTTACTTGTACAGCTCGTC
OEP7-SalI-F	AACCAATTCAGTCGACATGGGAAAAACTTCGGGA
OEP7-linker-R	ACCGCCGCTACCGCCGTCATCGGGGTCTTTGGT
linker-mVenus-F	GGCGGTAGCGGCGGTATGGTGAGCAAGGGCGAG
